# Harnessing the Potential of Enzymes as Inhaled Therapeutics in Respiratory Tract Diseases: A Review of the Literature

**DOI:** 10.3390/biomedicines10061440

**Published:** 2022-06-17

**Authors:** Gilles Vanderstocken, Nicholas L. Woolf, Giuseppe Trigiante, Jessica Jackson, Rory McGoldrick

**Affiliations:** 1 Enzybel Group SA, Drève Richelle 161-4 BAT P, 1410 Waterloo, Belgium; gvs@enzybel.com; 2 Inspira Pharmaceuticals Limited, 27 Old Gloucester Street, London WC1N 3AX, UK; nick@inspiraph.com (N.L.W.); drjessicajackson01@gmail.com (J.J.); 3 Centre for Cell Biology and Cutaneous Research, Blizard Institute, Queen Mary University of London, London E1 2AT, UK; g.trigiante@qmul.ac.uk

**Keywords:** enzyme therapeutic, botanical enzymes, respiratory tract disease, inhalation therapy, plant enzymes, plant protease, inhaled enzymes, respiratory tract infection, SARS-CoV-2, COVID-19

## Abstract

Respiratory tract diseases (RTDs) are a global cause of mortality and affect patient well-being and quality of life. Specifically, there is a high unmet need concerning respiratory tract infections (RTIs) due to limitations of vaccines and increased antibiotic resistance. Enzyme therapeutics, and in particular plant-based enzymes, represent an underutilised resource in drug development warranting further attention. This literature review aims to summarise the current state of enzyme therapeutics in medical applications, with a focus on their potential to improve outcomes in RTDs, including RTIs. We used a narrative review approach, searching PubMed and clinicaltrials.gov with search terms including: enzyme therapeutics, enzyme therapy, inhaled therapeutics, botanical enzyme therapeutics, plant enzymes, and herbal extracts. Here, we discuss the advantages and challenges of enzyme therapeutics in the setting of RTDs and identify and describe several enzyme therapeutics currently used in the respiratory field. In addition, the review includes recent developments concerning enzyme therapies and plant enzymes in (pre-)clinical stages. The global coronavirus disease 2019 (COVID-19) pandemic has sparked development of several promising new enzyme therapeutics for use in the respiratory setting, and therefore, it is timely to provide a summary of recent developments, particularly as these therapeutics may also prove beneficial in other RTDs.

## 1. Introduction

**Figure 1:**
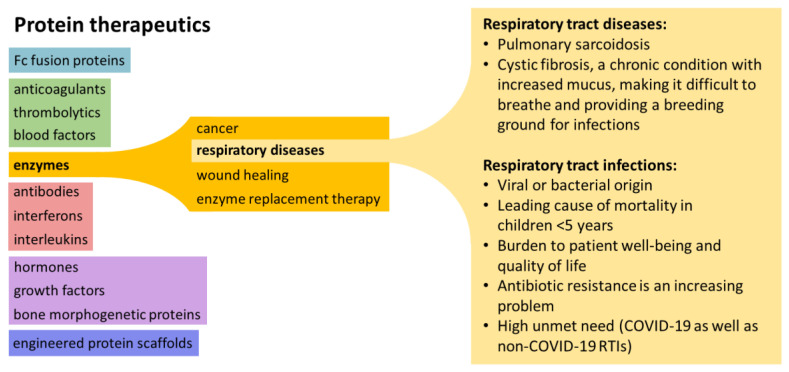
Schematic showing the scope of protein therapeutics and stratification to demonstrate the rationale for this review’s focus on enzyme therapeutics in respiratory diseases. COVID-19, coronavirus disease 2019; RTI, respiratory tract infection.

Enzyme therapeutics fall under the broader category of protein therapies, a diverse set of pharmacological agents also including molecules such as antibodies, anticoagulants, blood factors, cytokines, and hormones (Figure 1) [1]. Protein therapeutics typically exhibit an exceptionally high specificity for the target, ensuring a lower risk of interfering with normal biological processes. This results in a relatively good safety profile and complex functionality which can be difficult to replicate with small molecule drugs [1].

Protein therapeutics have been successful in treating conditions that would otherwise lack effective therapeutic options, including immune disorders, metabolic disorders, and certain cancers [1,2,3]. Despite the progress in protein therapeutics, the field is still considered to be in its infancy and in particular, the potential of enzyme therapeutics remains an untapped resource in drug discovery [3,4]. Enzymes for therapeutic use can have human, non-human animal, plant, or microbial origins [5,6,7,8]. At present, enzymes in medicine are mainly used to replace absent or faulty endogenous enzymes (enzyme replacement therapy [ERT]), making human enzymes the natural first choice. However, the therapeutic potential of enzymes extends beyond replacement therapy. Other therapeutic opportunities include uses in cancer, wound healing, microbial infections, and respiratory diseases with the option to administer the enzymes via various routes: orally, subcutaneously, intravenously, intrathecally, intracerebroventricularly, or via inhalation [9,10].

Plant-based enzymes, and other bioactive plant compounds, represent an area of interest in the development of biomedical applications as they can perform novel functions and/or function under conditions where human enzymes cannot [6]. The interest in enzyme therapeutics has increased in recent years, with the number of publications growing exponentially. Furthermore, ~30 new enzyme drugs were approved by the European Medicines Agency (EMA) between 2000 and 2020 versus fewer than five in the 20 years before that [4]. Table 1 provides a list of all enzyme therapeutics approved by the United States Food and Drug Administration (FDA) and/or the EMA.

There is presently considerable interest in the use of enzymes for the treatment of respiratory tract diseases (RTDs), particularly those exacerbated or caused by infections. The World Health Organisation (WHO) recognises reducing and preventing microbial infections as an urgent health priority for the next decade [28]. Vaccines have certain limitations, and the treatment of bacterial infections has relied largely on antibiotics, which are increasingly limited by antibiotic resistance [29]. Novel and effective therapeutic options for infectious respiratory diseases are an unmet need of particular importance and relevance, especially given the ongoing coronavirus disease 2019 (COVID-19) pandemic. Respiratory tract infections (RTIs) of the lower airways are classed as the world’s most deadly communicable disease and are listed fourth in the global leading causes of mortality, accounting for 2.6 million deaths in 2019 [30]. These infections can have a viral or bacterial origin and are a leading cause of mortality in children under 5 years old [30]. Additionally, chronic, recurrent, or severe respiratory infections can contribute a significant burden to patient well-being and quality of life [31,32,33]. 

While modern medicine has superseded more traditional, plant-based products for the treatment of bacterial, fungal, or viral infections [34,35], increasing levels of drug resistance are driving research into alternative approaches. In particular, the treatment of certain chronic RTDs, and frequently occurring RTIs, represents a large unmet medical need. The use of herbal extracts—including plant-based enzymes—in such indications warrants further exploration.

This review aims to summarise the current state of enzyme therapeutics in medical applications, with a focus on their potential to improve outcomes in RTDs, including RTIs. The rationale, challenges, current uses, and delivery mechanisms for enzyme therapeutics are also discussed alongside their application in the respiratory setting. Clinical developments in enzyme therapeutics are included to provide insights for future research in RTDs. 

## 2. Review Methodology

A narrative review approach was chosen to collate key evidence across a range of enzyme therapeutics in current use and development. To generate a comprehensive list of relevant publications and studies for potential inclusion, PubMed and clinicaltrials.gov were searched. Search terms included: enzyme therapeutics, enzyme therapy, inhaled therapeutics, botanical enzyme therapeutics, plant enzyme, herbal extract, and subsequent searches focused on RTDs, RTIs, and uses of enzymes in RTDs. See Table S1 for the full list of search terms.

## 3. The Rationale for Enzyme Therapeutics 

Enzymes play an essential role in all human metabolic processes, from digestion of food to highly complex immune responses, providing a vast range of avenues for research into harnessing their biocatalytic activity for therapeutics [36]. Enzymes generally have low toxicity coupled with a unique substrate specificity and, as biocatalysts, can be used to catalyse various processes including oxidation–reduction, hydrolysis, isomerisation, and proteolysis [37]. 

### 3.1. Enzymes Derived from Animals

**Figure 2:**
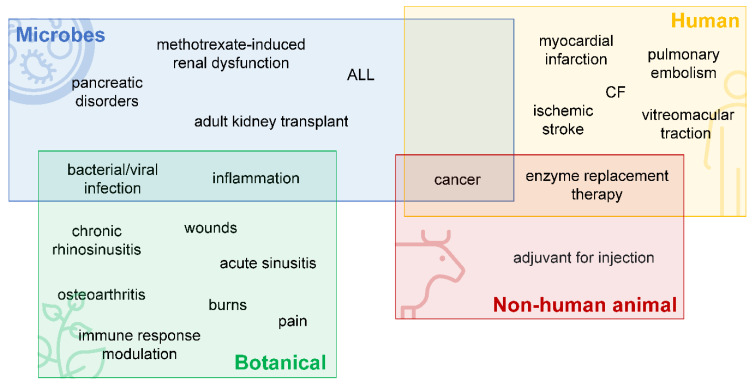
Graphic representations of the different origins for enzymes used in enzyme therapeutics with their current/potential uses listed. Microorganisms, plants, non-human animals, and humans are all sources for enzymes in therapeutics. Enzyme therapies are currently available to treat a wide variety of diseases, including cancers, infections, wounds, and as enzyme replacement therapy for metabolic diseases. NB, this figure represents the information described in this review and is not intended to provide an exhaustive list. ALL, acute lymphoblastic leukaemia; CF, cystic fibrosis.

A concern when developing drugs is the potential antigenicity of new treatments. To reduce this risk, human or non-human animal biologics are the first choice when looking for enzymes to exploit. Recombinant mammalian enzymes are currently used to treat numerous diseases, performing a variety of different functions such as tissue plasminogen activation, or hydrolysis of collagen or DNA [22] (Figure 2). Additionally, ERT uses the action of exogenously produced enzymes to compensate for a deficit of a specific enzyme in the case of genetic disorders or where required as a result of complications from other conditions. Examples where ERT is considered a first-line treatment option are lysosomal storage diseases, exocrine pancreatic insufficiency [9], and irritable bowel syndrome [38,39,40]. The supplementation of native enzymes is also used in the treatment of RTIs. For example, while lysozyme is normally present in airway secretions to prevent bacterial growth, additional administration of this enzyme can enhance levels of protection [41].

### 3.2. Enzymes Derived from Microbes

Bacteria, fungi and yeast are all potential sources of therapeutic enzymes, and currently, microbial enzymes are used in indications such as cancer, pancreatic disorders, bacterial or viral infections, and inflammatory diseases [8] (Figure 2). As therapies, microbial enzymes are advantageous in that they are relatively cheap and easy to manufacture at a large scale [42] and possess fewer ethical concerns compared with enzymes of animal origin [43]. Viruses are another microbial source of therapeutic enzymes. For example, the bacteriophage-derived ‘enzybiotics’ are capable of degrading bacterial cell walls to control pathogen populations [44]. More broadly, phage enzymes with promising antimicrobial properties include lysins, autolysins, lysozymes, bacteriocins, endolysins, and depolymerases [42,45,46,47], although there are practical hurdles when developing these for in vivo application [48]. Currently, several enzybiotics are in clinical development, such as the endolysin CF-301 to treat *Staphylococcus aureus* blood stream infection and endocarditis. When combined with standard-of-care antibiotics, this treatment showed a higher clinical response rate in the subgroup of people with methicillin-resistant *Staphylococcus aureus*, compared with antibiotics alone [49]. Phage lysins are also being developed specifically for RTIs [50] and the modularity of some endolysins makes them amenable to creating chimeric forms with multiple activities such as enzymatic action and cell-wall binding [51].

### 3.3. Enzymes Derived from Botanical Sources

Plants can be a source for various kinds of enzymes, including lipases, amylases and proteases [52]. Enzymes from this source represent an underutilised area in enzyme therapeutics and few treatments are currently available. Compared with human enzymes, many plant proteases display higher stability and a wider pH and temperature range for enzymatic activity [6]. This is because plant proteases often exist as a cocktail of multiple different enzymes. For example, papain (a commercial product extracted from the latex of papaya) is a mixture composed of the following cysteine proteases: papain (the molecule of the same name), chymopapain, glycyl endopeptidase and caricain [53,54]. Papain and chymopapain were also found in papaya peels, and it is suggested the peels contain additional, new proteases [55]. Bromelain, extracted from pineapple, consists of the enzymes bromelain, ananain, and comosain [56].

Enzymes derived from plant-based sources are generally considered to be safe; however, plants can contain substances toxic to human health, necessitating appropriate purification and testing of raw extracts to ensure safety prior to use. For example, papaya extracts have analgesic, anti-inflammatory and immunomodulatory activity [57,58,59], and the purified enzymes have been investigated for use in wound healing and have been used for the treatment of intervertebral disc herniation (Figure 2) [60,61]. However, the raw papaya latex from which most papain is sourced can be associated with skin sensitivity, asthma, eye irritation, and anaphylactic reactions [62]. Latex allergies are relatively common in the general public (4.3%) and more prevalent in those exposed to latex as part of their job (9.7%) or as patients in a healthcare setting (7.2%) [63].

Evidence suggests some plant enzyme extracts possess antitumour, antibacterial and/or antifungal activity and extracts have been used on a small scale for the treatment of delayed wound healing, burn debridement, and osteoarthritis [6,64,65,66]. Other extracts have been shown to possess activity against coronaviruses, mediated through inhibition of viral entry and/or replication, or through an as-yet unknown antiviral mechanism [67]. In the respiratory setting, bromelain has demonstrated good distribution to serum and rhinosinusal tissues when administered as a tablet in people with chronic rhinosinusitis showing relief of symptoms [68,69]. In children with acute sinusitis, bromelain was associated with a faster recovery from symptoms without side effects (except in one participant with a pineapple allergy) when compared with children receiving one of the combination therapies (bromelain + standard treatment) and children receiving standard treatments only [70]. A hypersensitive reaction to inhalation of plant enzymes after long-term occupational exposure has been previously described [71] and possible immunological cross reactivity of plant enzymes should be considered. Bromelain is also used as a complementary therapy in nasal and sinus swelling and, due to its anti-inflammatory effects, has been used in many non-respiratory conditions [65].

Bromelain and papain were both investigated in a study that conjugated each enzyme to nanoparticles to establish whether coated nanoparticles could function as a mucus-permeating drug delivery system. Results showed that both enzymes demonstrated mucus-permeating functionality, with bromelain showing better activity than papain [72]. The results suggested that these enzymes could be exploited for their mucus-clearing properties and also co-administered with other therapeutics to aid delivery to sites with increased mucus.

The biosynthetic pathways are known for less than 0.1% of all metabolites thought to exist in plants. Recent research that created a computational pipeline to identify metabolic enzymes, pathways, and gene clusters predicted over 150,000 enzymes from 22 species (21 plant species, one algal species) [73], showing the large potential for discovery of novel plant enzymes that may have clinically useful functionality.

### 3.4. Challenges with Enzyme Therapeutics

The main challenges with enzyme therapeutics are in maintaining functional ability, prolonging activity to ensure a sustained presence inside the body, tissue specificity and immunogenicity [4,74]. For example, the short half-life of some proteases is generally a disadvantage for their therapeutic application because it requires the protease to be dosed frequently [75]. Modifications to therapeutic enzymes or their delivery mechanism can increase this half-life, such as adding a polyethylene glycol moiety to the therapeutic enzyme and additionally delivering it via carrier erythrocytes [76]. Enzymes can be highly sensitive to changes in their physical and chemical environment and may be unable to function at the intended site of action. Another challenge is the lack of tissue specificity and ensuring enzyme activity does not pose a risk to the surrounding healthy tissue. A drug-induced immune response—the production of antidrug antibodies that might be accompanied by a fatal reaction or development of autoimmunity—must be monitored as this can compromise the efficacy and safety of a therapy. Enzyme encapsulation has been investigated as a possible solution for poor tissue specificity as well as to reduce clearance and immunogenicity [4]. Practical concerns can also apply. For example, the enzyme formulation must be uncontaminated with other substances (such as protease inhibitors) that might affect the reaction. Similarly, inappropriate or long-term storage might result in degradation of active ingredients, particularly as proteases are susceptible to autolytic degradation.

Despite the practical concerns, a Global Market Insights report predicts significant growth in the use of enzymes as therapeutics with a predicted global market value for proteases of around US$ 2 billion by 2024 [77]. A driver for this predicted growth is the anticipated increase in the use of proteases as therapeutics [37].

## 4. Inhaled Enzyme Therapeutics to Treat RTDs

The use of enzymes in the treatment of RTDs involves three considerations which must be balanced: (1) the direct targeting of the site of illness/infection; (2) the reduced reliance on antibiotic(s) in case of infection; and (3) the stabilisation of the enzyme formulation [78]. Protein therapeutics have been conventionally administered via a systemic route, but this can be associated with side effects and is an inefficient method for drug delivery in RTDs, where locally acting agents are required at the affected site (typically the lungs and/or nasal cavity) [74]. Although systemic enzyme therapy, administered orally, has been explored to treat patients with chronic airway diseases [79,80,81], pulmonary delivery can ensure high drug concentrations at the affected site with a rapid clinical response [82,83]. Inhalation is therefore the preferred route of administration in the treatment of common respiratory diseases [84]. In the context of RTIs, where there is a heavy reliance on antibiotics to treat bacterial infection, another avenue of considerable interest is inhaled drugs to tackle the formation of biofilms. Biofilms are complex structures of bacteria and other materials that adhere to each other and to surfaces, such as the lining of the respiratory tract [85]. Biofilms contribute to chronic infection as they encase the underlying bacterial cells, leading to a much higher tolerance to antibiotics [86]. Biofilm formation hinders the ability of antibiotics and the immune system to eradicate infection and, especially in the respiratory tract, can be difficult to reach with therapeutic agents. Inhaled antibiotics can be effective in reaching and treating RTIs but do not target the biofilm itself and require higher doses to penetrate the biofilm. There are currently no effective treatments for biofilms and the issue demands novel responses that do not rely on antibiotics [87,88]. Here, enzyme therapeutics hold potential and could be developed to degrade existing biofilms and/or help prevent their initial formation in cases of chronic infection. Several enzyme candidates are now in the early stages of investigation as anti-biofilm agents [89,90].

### 4.1. Advantages and Disadvantages of Inhaled Therapies

The pulmonary system is a well-established route of entry for many active pharmacological agents and is commonly used to treat RTDs such as asthma and chronic obstructive pulmonary disease. Inhalation can deliver relatively high doses of a drug directly to the affected site while minimizing systemic exposure and side effects [91]. However, drug inhalation comes with some challenges (Table 2) that can reduce the effective concentration of active ingredient [74]. To combat these issues, additional pharmacokinetic considerations—such as formulation stability, retention and clearance, and dose regulation—are required when designing protein-based therapies for inhaled use [74]. Moreover, physical characteristics of the administered form of the drug, such as viscosity, conductivity, osmolality, pH and particle size, need to be considered too as they affect in which parts of the airways the drug will be deposited and can be absorbed and tolerated [82,92,93]. Finally, the inhalation device used for delivery is a crucial aspect and must be used correctly and easily by the patient for effective inhalation.

Another practical concern is the use of excipients, as only a few have been approved for inhalation [74]. Excipients are compounds that are often added to the drug formulation to improve performance by reducing clearance, enhancing stability and solubility, and/or preventing aggregation. Airway tolerability of non-approved excipients can be assessed relatively quickly early in a development programme, enabling poorly tolerated excipients to be excluded from further evaluation. An alternative way to overcome some of the challenges of inhalation therapy is more frequent dosing; however, this may lead to reduced adherence to treatment [97]. Moreover, chronic inhalation with protein/peptide-based therapies, especially proteases, may lead to local immunogenicity or irritation to the airways [83,96].

Despite the challenges, significant progress has been made in the design of protein therapeutics for inhaled delivery and several locally and systemically acting protein therapeutics have been successfully developed [84,98]. Inhalation technology has also improved rapidly in recent years in recognition of the practicality and efficiency of the route of administration [99]. Many protein therapeutics now use custom nebulisation devices tailored to the active ingredient and this might be one of the ways in which inhaled enzyme therapeutics can be further improved.

### 4.2. Current Uses of Inhaled Enzyme Therapeutics in RTDs

To date there is only one enzyme therapy approved for inhaled delivery to treat a respiratory condition: dornase alfa (Pulmozyme^®^). This is mucolytic deoxyribonuclease (recombinant human DNase [rhDNase I]) that is currently used for treatment of patients with cystic fibrosis (CF) and is delivered via inhalation. Approved by the FDA in 1993 [20], the enzyme breaks up DNA and reduces the viscosity of lung secretions facilitating mucus clearance [10,21,100]. Alidornase alfa (PRX-110, AIR DNase^TM^) is a human DNase I modified to resist inhibition by actin and received orphan drug designation for pulmonary sarcoidosis by the FDA in 2020 [101]. In a clinical trial, alidornase alfa was well tolerated in people with CF, showing only mild adverse events and is now being developed for other indications [102].

To our knowledge there are no inhaled botanical enzymes currently used for the treatment of RTDs or RTI.

## 5. Developing Enzyme Therapeutics for RTD

**Figure 3:**
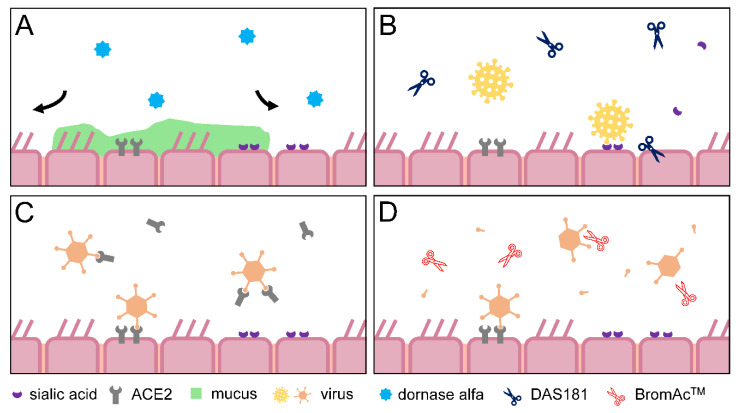
Schematic of proposed mechanisms of action for inhaled enzyme therapeutics in the respiratory tract. (**A**) dornase alfa cleaves extracellular DNA, reducing the viscosity of mucous and making it easier for the airways to clear the mucus. (**B**) DAS181 catalyses sialic acid cleavage from glycoproteins and glycolipids present on the lung epithelium, which interferes with the lifecycle of some viruses. (**C**) the ACE2 receptor is used by the SARS-CoV-2 virus to enter cells and administering ACE2 is expected to act as a decoy for the virus, preventing infection. (**D**) BromAcTM is purified bromelain combined with N-Acetyl Cysteine and is thought to break down the spike protein of the SARS-CoV-2 virus thereby eliminating the ability of the virus to infect cells. Abbreviations: ACE2, angiotensin converting enzyme 2; DNA, deoxyribonucleic acid.

Several enzyme therapies are currently being developed for use in RTDs and to combat RTIs via different mechanisms of action (Table 3, Figure 3).

### 5.1. Dornase Alfa

Dornase alfa, approved for use in CF, is now being investigated for use in COVID-19 and has been reported as well-tolerated when co-administered with albuterol via a nebuliser to mechanically ventilated patients with COVID-19 [108]. Albuterol is a short-acting β_2_ adrenergic receptor agonist that relaxes the smooth muscle of the airways to further improve delivery to the alveoli [108]. Dornase alfa may reduce the oxygen requirements of ventilated patients with COVID-19 by reducing the viscosity of mucoid sputum. As a result of these findings several clinical trials are now underway to test dornase alfa in COVID-19 (NCT04359654, NCT04432987, etc.) and also in other conditions such as acute respiratory distress syndrome (NCT04402970) and ischemic stroke (NCT04785066).

### 5.2. DAS181

Nebulized DAS181 (Fludase^®^) is in Phase 3 development for the treatment of parainfluenza (NCT03808922) and in Phase 2 trials to treat a number of other viruses that depend on sialic acid receptors. DAS181 is a host-directed enzyme-based drug with sialidase activity derived from bacteria, which catalyses sialic acid cleavage from glycoproteins and glycolipids in the lung epithelium thereby interfering with the life cycle of certain viruses [104]. DAS181 was recently investigated for safety in a small open-label trial for compassionate use in hypoxic patients with severe COVID-19 and demonstrated no treatment-related adverse events [109]. Nebulized DAS181 was investigated in a study on immunocompromised adults with parainfluenza virus lower RTI who required oxygen supplementation. A larger proportion of patients who received DAS181 showed clinical improvement compared with placebo. However, the difference was only significant in a post-hoc analysis of patients who had a history of solid organ transplant, or a recent history of haematopoietic cell transplantation or chemotherapy, and who required supplemental oxygen [110].

### 5.3. Angiotensin Converting Enzyme 2

A recombinant human angiotensin-converting enzyme 2 (rhACE2) is in development for several respiratory diseases, such as acute lung injury and pulmonary arterial hypertension. ACE2 is the cellular entry receptor for SARS-CoV-2 and its receptor-binding domain on the virus presents a potential target for antiviral treatment [111]. Alunacedase alfa (APN01), an rhACE2 that may be able to block viral entry by acting as a decoy, is being investigated for inhaler delivery to people with newly diagnosed COVID-19 who have co-morbidities or are elderly. A Phase 2 trial is underway in patients with COVID-19 using APN01 as an intravenous treatment (NCT04335136), and a Phase 1 trial is scheduled to investigate APN01 as an inhaled therapy for COVID-19 (NCT05065645).

### 5.4. Other Pipeline and Pre-Clinical Therapeutics

A review published in 2020 suggested bromelain as a potential treatment for symptoms of COVID-19 because of its anti-inflammatory and anticoagulatory properties, especially when combined with antiviral drugs [112]. BromAc^TM^, a formulation of purified bromelain combined with N-Acetyl Cysteine, is thought to degrade the spike protein of the SARS-CoV-2 virus, thereby eliminating the virus’ ability to infect cells. The drug is planned to be administered nasally and is currently undergoing trials with the aim to help reduce the impact of the virus in people with respiratory complications [106,107]. Bromelain is also scheduled to be investigated in a study that evaluates zinc, vitamin C, and the plant pigment quercetin co-administered orally in patients with COVID-19 in a Phase IV trial (NCT04468139) which is currently recruiting participants.

A novel botanical extract of proteases (IPX), derived from *Carica papaya*, has shown virucidal properties in vitro and, in other preliminary in vitro experiments, inhibited recombinant ACE2 receptors from binding to the SARS-CoV-2 spike protein [89]. Further formulation developments aim to stabilise and maintain the enzymatic activity in an aqueous formulation suitable for inhalation and to increase the potency of the enzyme extracts. In vivo efficacy and safety are yet to be determined, but trials to explore its potential in COVID-19 and other RTIs are planned. Additionally, IPX is being investigated for its potential to degrade biofilms. Another enzyme under investigation for its ability to combat biofilms is pyocyanin demethylase (PodA), an enzyme that converts an extracellular metabolite of *Pseudomonas aeruginosa* and may prevent formation of biofilms. The combined use of PodA and antibiotics has been demonstrated to increase the killing of recalcitrant *P. aeruginosa* cultures in biofilms [90].

Another strategy to treat RTIs is to locally administer specific enzymes naturally present in the lungs to reach supraphysiological concentrations. In one round of experiments, a charge-variant lysozyme reduced inflammatory markers and lung injury [113] and was found to have antiviral and immunomodulatory properties. Lysozyme has since been proposed as a treatment for COVID-19 (albeit not via inhalation but via oral administration) [114]. Lactoferrin and lactoperoxidase are two other enzymes normally present in the lungs [115]. Lactoferrin is scheduled to be investigated in COVID-19 in multiple clinical trials, and lactoperoxidase function has been suggested as a possible factor in defence against viruses including SARS-CoV-2 [116]. The search for effective treatment in COVID-19 is also exploring oral administration of enzymes, such as the serine-protease serratiopeptidase as an adjuvant for the management of COVID-19 [117]. In an open-label pilot study using enzyme supplements to treat pulmonary fibrosis, improvements in symptoms, mental and physical wellbeing, and in health-related quality of life compared with the baseline were observed [118]. Research into the oral use of enzyme supplements in RTDs highlights another potential opportunity for enzyme therapeutics in the respiratory setting.

## 6. Conclusions

Enzyme therapeutics benefit from an exceptionally high substrate specificity, low systemic toxicity, and ability to catalyse a range of reactions. The use of enzymes is an established treatment for certain inherited and rare diseases. However, there is a large unmet need for novel treatments to a range of respiratory conditions, for which new enzyme therapies—particularly from underexplored resources—might offer significant potential. Triggered by the COVID-19 pandemic, several promising enzyme therapeutics are currently in development for RTDs and may soon be an adjuvant or replacement for conventional antiviral or antimicrobial treatments.

## Figures and Tables

**Table 1:** FDA/EMA approved enzyme therapeutics for use in humans, currently available. Drugs are listed as type of enzyme (underlined), generic drug name, and trade name (in brackets). Enzyme therapies are listed with their approved indications, indications currently being investigated in clinical trials, and the therapeutic’s mechanism of action.

Year Approved ^a^	Drug (Brand Name)	Approved Indication(s); *Clinical Trials If Applicable*	Mechanism of Action	Reference(s)
FDA: Not approved EMA: 2020	**Cysteine protease** **Imlifidase** **(Idefirix^®^)**	Desensitization treatment in highly sensitized adult kidney transplant (EMA only and currently in clinical trials in the USA) *In clinical trials for: GBS, AMR pre-treatment in DMD and LGMD (to enable gene therapy), and anti-GBM antibody disease*	The enzyme is derived from *Streptococcus* pyogenes and works by degrading IgG antibodies	Idefirix^®^ product website [11] Hansa Biopharma and Sarepta Therapeutics agreement [12]
FDA: 2012 EMA: 2022	**Glutamate carboxypeptidase** **Glucarpidase (Voraxaze^®^)**	Methotrexate-induced renal dysfunction *In clinical trials for: Certain types of cancer, including lymphoma, osteosarcoma, and leukemia*	Methotrexate hydrolysed to glutamate and less toxic 2,4- diamino-N10-methyl-pteroic acid largely excreted by the liver	Voraxaze^®^ product website [13]
FDA: 2012 EMA: 2013	**Microplasmin** **Ocriplasmin** **(Jetrea^®^)**	Vitreomacular traction *In clinical trials for: exudative age-related macular degeneration, vitreomacular traction/adhesion etc.*	Active against fibronectin and laminin, components of the vitreomacular interface. Enzyme dissolves proteins that link the vitreous humour to the retina	Jetrea^®^ SmPC [14]
FDA: Not approved EMA: 2012	**Enzyme mixture including bromelain** **NexoBrid^®^**	Removal of eschar from deep partial-thickness and full-thickness burns of the skin caused by heat or fire *In clinical trials for: (thermal) burns*	Concentrate of proteolytic enzymes enriched in bromelain	NexoBrid^®^ product website [15]
FDA: 2005 EMA: Not approved	**Hyaluronidase ** **(Amphadase^®^; Hylenex Hylenex^®^; Vitrase^®^)**	Adjuvant to increase absorption/dispersion of other injected drugs; hypodermoclysis; as an adjunct in subcutaneous urography for improving resorption of radiopaque agents *In clinical trials for: combination therapy for different cancers, combination therapy for CIDP and MMN*	Degradation of hyaluronic acid (a main component of extracellular matrix)	Amphadase^®^ prescribing information [16]
FDA: 2002 EMA: 2001	**Urate hydroxylase** **Rasburicase** **(Elitek^®^; Fasturtec^®^)**	Management of plasma uric acid levels during anticancer therapy *In clinical trials for: Other types of cancer, including leukemia, lymphoma and tumor lysis syndrome*	Recombinant urate-oxidase enzyme produced by a genetically modified *Saccharomyces cerevisiae* strain. Converts uric acid to allantoin in patients with hyperuricemia. Soluble allantoin is excreted via the kidneys	Elitek^®^ product website [17]
FDA: approx. 1999 and later EMA: approx. 2001 and later	**Various enzymes for cancer**	Various cancers	Nutrient deprivation Remodelling of the fibrotic tumour microenvironment Management of tumour lysis syndrome Inhibition of protein synthesis	Cioini et al. 2022 [18]
FDA: 1996 EMA: 1996	**Tissue plasminogen activator** **Reteplase** **(Retavase^®^; Rapilysin^®^)**	Suspected heart attack to dissolve blood clots (use within 12 h) *In clinical trials for: myocardial infarction*	Activates production of plasmin, which breaks up blood clots	Retevase^®^ product website [19]
FDA: 1993 EMA: 1994	**DNase** **Dornase alfa (Pulmozyme^®^)**	CF *In clinical trials for: ARS, ARDS in COVID-19*	Mucolytic that cleaves extracellular DNA, reducing mucus viscosity	US FDA [20] Genentech [21] Lazarus et al. 2019 [10]
FDA/EMA: 1990 and later	**Various enzymes for ERT**	Enzymes for ERT, most are developed to treat inborn errors of metabolism	ERT to make up for a missing or defected native enzyme	Baldo et al. [22]
FDA: 1987 EMA: 2002	**Tissue plasminogen activator** **Alteplase** **(Activase^®^; Actilyse^®^; Cathflo^®^)**	Myocardial infarction with ST elevation; acute ischemic stroke; pulmonary embolism *In clinical trials for: kidney disease and certain types of stroke*	The recombinant tissue plasminogen activator binds fibrin in the thrombus and cleaves a specific bond in plasminogen which creates plasmin, causing local fibrinolysis	Actilyse^®^ product website [23]
FDA: 1978 EMA: 2016 FDA: 1994 EMA: 2016 FDA: 2018 EMA: Not approved	**Asparaginase ^b^ ** **Crisantaspase** **Pegaspargase** **Calaspargase pegol**	ALL (in combination with other drugs) *In clinical trials for: lymphoma, multiple myeloma, other types of leukemia*	Contains asparaginase that reduces blood levels of asparagine, an amino acid that healthy cells can produce and cancer cells cannot, resulting in cancer cell death	Elspar^®^ highlights of prescribing information [24] Oncaspar product website [25] Asparlas^TM^ prescribing information [26]

^a^ The year of approval for the first drug in that class is listed, and in some cases, this is an approximation. ^b^ There are three different types of asparaginase approved for use in ALL, differing in their source/expression system and without/with modification to the molecule. Only the generic names are included in the table. The following asparaginases are commercially available: Erwinase^®^/Erwinaze^®^, Rylaze^TM^, Elspar^®^, Spectrila^®^, Kidrolase, Leunase^®^, Oncaspar, Asparlas^TM^. Table adapted from: Kinch et al. 2015 [27]; Baldo et al. 2015 [22]; Cioni et al. 2022 [18] and supplemented with therapies approved from 2015 until February 2022 inclusive, found on the FDA and EMA websites. Information on indications in clinical trials is from clinicaltrials.gov, accessed on 30 March 2022. Drugs withdrawn since approval have not been included. ADA, adenosine deaminase; ALL, acute lymphoblastic leukaemia; AMR, antibody mediated rejection; ARDS, acute respiratory distress syndrome; CF, cystic fibrosis; CIDP, chronic inflammatory demyelinating polyradiculoneuropathy; EMA, European Medicines Agency; DMD, Duchenne muscular dystrophy; ERT, enzyme replacement therapy; FDA, United States Food and Drug Administration; GBM, glomerular basement membrane; GBS, Guillain-Barré syndrome; IM, intramuscular; IV, intravenous; LGMD, limb-girdle muscular dystrophy; MMN, multifocal motor neuropathy; SC, subcutaneous, SmPC, summary of product characteristics; USA, United States of America.

**Table 2:** Advantages and challenges of inhaled therapies for RTDs.

Advantages	Challenges
Established delivery method for existing approved drugs	Drug not reaching intended site of action due to clearance mechanisms or degradation/aggregation [94]
Ability to deliver high doses of drug directly to the site where it is needed [91]	Drug metabolised too quickly or dissolution upon reaching the lungs [94]
Minimizes systemic exposure and systemic side effects [91]	Potential formation of antidrug antibodies (ADAs) [95] which can affect pharmacokinetics, efficacy, and lead to severe adverse events [74]
Rapid clinical action	Only a few excipients (needed for formulation stability etc.) have been approved for inhalation [74]
Inhalation devices can be used at home by the patient, avoiding the need for a hospital visit	Chronic inhalation with protein/peptide-based therapies may lead to local immunogenicity or irritation to the throat [83,96]
	Difficulty in ensuring therapy has properties suitable for inhalation such as pH, osmolality, viscosity, and appropriate droplet size
	Requirement for development of treatment-specific devices, particularly if agent is unstable

RTD, respiratory tract disease.

**Table 3:** Inhaled enzyme therapeutics for respiratory conditions currently in development.

Molecule (Drug Name); Company	In Development for: Indication (Phase, Trial Number)	Mechanism of Action (MoA)	Reference(s)
Deoxyribo-nuclease I: Alidornase alfa (AIR DNase^TM^, PRX-110); Protalix	In development for: CF (Phase 2, NCT02722122),	Mucolytic that cleaves extracellular DNA, reducing mucus viscosity	US FDA [101] Protalix [102]
Deoxyribo-nuclease I: rhDNase	In development for: neutrophilic asthma (Phase 1/2, NCT03994380)	Mucolytic that cleaves extracellular DNA, reducing mucus viscosity	Lazarus et al. 2019 [10]
Alunacedase alfa (rhACE2; APN01);	In development for: acute lung injury, pulmonary arterial hypertension; COVID-19 (Phase 1, NCT05065645)	ACE2 is the cellular receptor used by some viruses to enter the cells. Administering rhACE2 is expected to function as a decoy for viruses to block viral entry	Apeiron Biologics press release 2020 [103]
Sialidase (Fludase, DAS181); Remin Hospital of Wuhan University; Ansun Biopharma	In development for: severe COVID-19 (Phase N/A, NCT04324489) lower tract parainfluenza infection (Phase 3, NCT03808922) non-IFV rhinovirus infections (Phase 2, NCT04298060)	Catalyses sialic acid cleavage from glycoproteins and glycolipids in the lung epithelium, which interferes with the lifecycle of some viruses	Chan et al. 2009 [104] PRNewswire press release [105]
BromAc^TM^; mucpharm	In development for: COVID-19 with respiratory complications	Degrades the spike protein of the SARS-CoV-2 virus, rendering the virus unable to infect cells	Akhter et al. 2021 [106] mucpharm company website [107]
IPX formulation; Inspira Pharmaceuticals	In development for: COVID-19 and other RTIs	MOA not yet known	Inspira Pharmaceuticals press release (2021) [89]

ARDS, acute respiratory distress syndrome; CF, cystic fibrosis; COVID, coronavirus disease; IFV, influenza virus; rhACE2, recombinant human angiotensin-converting enzyme 2; RTI, respiratory tract infection.

## Data Availability

Not Applicable.

## Supplementary Materials

The following supporting information can be downloaded at: https://www.mdpi.com/article/10.3390/biomedicines10061440/s1, Table S1: title: Search strings used in the search for literature using PubMed and the search for clinical trials using ClinicalTrials.gov.
