# Supplementary material for: Harnessing the Potential of Enzymes as Inhaled Therapeutics in Respiratory Tract Diseases: A Review of the Literature
Source: Biomedicines. 2022 Jun 17;10(6):1440. doi: 10.3390/biomedicines10061440 (PMC9220205; doi:10.3390/biomedicines10061440)
Supplement: Supplementary file 1 [file biomedicines-10-01440-s001.zip › biomedicines-1682579-supplementary.pdf]

Supplementary Material

Table S1. Search strings used in the search for literature using PubMed and the search for clinical trials using ClinicalTrials.gov.

Database	Search terms
PubMed	(enzyme therap*) OR (inhaled therap*) OR (inhalation therap*) OR (enzyme drug) OR (botanical enzyme) OR (botanical drug) OR (herbal extract*) OR (plant-derived enzyme) OR (plant-derived drug) or (plant derived extract) OR (enzybiotics)<br><br>Filters: publications in humans, and in the English language.
	(respiratory infection) OR (infection) OR (respiratory tract infection OR RTI) OR (viral infection) OR (microbial infection) OR (respiratory disease)<br><br>Filters: publications in humans, and in the English language.
ClinicalTrials.gov	**Condition or disease:** (respiratory infection) OR (respiratory disease) OR (COVID-19 OR SARS-CoV-2) OR (respiratory syndrome) OR RTI<br><br>**Other terms:** (enzyme therapeutic) OR (enzyme therapy) OR (plant enzyme)<br><br>Filter: Terminated recruitment status<br><br>*Subsequent searches used Google to determine other pipeline drugs in development in RTIs and COVID-19 and the use of enzymes in RTI.*

COVID, coronavirus disease; RTI, respiratory tract infection.
